# Metronidazole-Induced Pancreatitis

**DOI:** 10.1155/2010/523468

**Published:** 2010-09-01

**Authors:** E. O'Halloran, A. Hogan, K. Mealy

**Affiliations:** Department of Surgery, Wexford General Hospital, Wexford, Ireland

## Abstract

*Case Summary*. A 25-year-old caucasian lady presented to the Accident & Emergency department complaining of acute onset severe epigastric pain radiating through to the back with associated nausea and vomiting. A diagnosis of acute pancreatitis was made. Symptoms commenced after the third dose of Metronidazole therapy prescribed for a recurrent periodontal abscess. The patient described a similar episode 10 months previously. On neither occasion were any other medications being taken, there was no history of alcohol abuse and no other gastro-intestinal aetiology could be identified on imaging. Symptoms resolved quickly upon discontinuation of the antibiotic agent. We conclude therefore that Metronidazole can reasonably be identified as the only potential causative agent. *Discussion*. The proportion of cases of pancreatitis caused by drugs is estimated to be around 2% in the general population. The exact mechanism of action of Metronidazole induced pancreatitis is unclear but a trigger role for the drug seems likely. *Conclusion*. This case provides the eighth report of Metronidazole induced pancreatitis. All of the cases were reported in females and ran a benign course.Early diagnosis, discontinuation of the drug and supportive care will lead to a successful recovery in the majority of cases.

## 1. Introduction

Metronidazole is a nitroimidazole antibacterial medication used mainly in the treatment of infections caused by anaerobic bacteria and protozoa. It is commonly known by the trade name Flagyl. Systemic Metronidazole is indicated for the treatment of many anaerobic infections which can clinically present as gynaecological conditions; bacterial vaginosis, and abdominal conditions; Crohn's disease, necrotizing pancreatitis and gastritis. Metronidazole is also commonly prescribed in the treatment of acute gingivitis and periodontal infections [1].

The adverse side effects of Metronidazole include gastrointestinal upset, metallic taste [2], urticaria [3], headache, peripheral neuropathy [2], and rarely pancreatitis. There are only seven reported cases of Metronidazole-induced pancreatitis in the English language literature to date (Table 1).

Acute pancreatitis is an inflammatory disease with a wide spectrum of severity [9]. It is clinically characterized by acute constant abdominal pain [10] and elevated pancreatic enzymes [10]. Alcoholism and biliary tract stone disease account for 95% of cases [11]. Acute pancreatitis is classified as mild, moderate, or severe, the differentiation of which is made by using disease severity scores such as Imrie [9], Ranson, and more recently APACHE II. 

We report a case of recurrent mild-acute pancreatitis, Imrie score I, associated with just 3 doses of Metronidazole therapy prescribed for a periodontal abscess.

## 2. Case Report

A 25-year-old caucasian lady presented to the Accident & Emergency Department of a general hospital complaining of acute onset severe epigastric pain radiating through to the back with associated nausea and vomiting. History, examination and biochemical analysis confirmed a diagnosis of acute pancreatitis, Imrie score 1 [9]. Symptoms commenced after the third dose of Metronidazole therapy prescribed by the patient's dentist for treatment of a periodontal abscess. Amylase was 810 on admission and CRP was 70. CRP peaked to 248 on day 4 and rapidly declined thereafter. Relative negatives in the history included no other concurrent medications, no known drug allergies, alcohol consumption of 3–6 units weekly, and the patient was a nonsmoker. 

Relevant past surgical history included; (1) laparoscopic cholecystectomy (June 2006) for symptomatic treatment of suspected biliary colic although gallstones were not identified on imaging and (2) first episode of acute pancreatitis requiring hospital admission (Septmber 2009) following 5 doses to Metronidazole therapy prescribed for dental abscess. Ultrasound scan, Computed Topography of abdomen, and Endoscopic Retrograde Cholangio-Pancreatography all failed to demonstrate gallstones as potential aetiology at that time. 

On this admission, a detailed history and biochemical evaluation excluded other risk factors for pancreatitis-gallstones, increased alcohol intake, recent ERCP, hypercalcaemia, hypercholesterolaemia, or viral infections. She was not taking any regular medications nor did she have any known drug allergies. Imaging by ultrasound scan reported previous cholecystectomy, normal common bile duct, and once again no gallstones were visualized. Magnetic Resonance Cholangio-Pancreatography also demonstrated normal intra- and extrahepatic biliary anatomy. These factors combined with past surgical history provided convincing evidence of Metronidazole as the most probable aetiological factor. 

Supportive management of IV fluid resusitation, analgesia, antiemetic and, nil per oral was instituted and Metronidazole therapy was immediately discontinued. Her clinical course was uncomplicated and amylase returned to normal on day 4 post admission (Table 2). She began to tolerate light diet on day 5 and was discharged on day 7.

## 3. Discussion

The proportion of cases of pancreatitis caused by drugs is estimated to be around 2% in the general population, with much higher proportions in high risk groups, such as children and HIV positive patients [12]. 

Specific criteria to support drug-induced illness was first described by Mallory et al. in 1980; case reports with the strongest evidence are those that (1) clearly diagnose pancreatitis and exclude common aetiologies, (2) provide the dose and time interval between the start of treatment with the suspected drug and the development of pancreatitis, (3) document response to withdrawal of the drug, and (4) demonstrate recurrent pancreatitis upon rechallenge with the drug [13]. The majority of cases are mild [14], but severe and even fatal cases of drug-induced pancreatitis may occur, thus making identification of the offending agent critical [12]. 

Although there are only 7 previous case reports of Metronidazole-induced pancreatitis in the English literature, the drug meets the above criteria and can be classified as having a probable association with the onset of acute pancreatitis [15, 16].

Our patient developed pancreatitis after the third dose of Metronidazole on two different isolated occasions 10 months apart. On neither occasion could any other aetiology be identified, in particular gallstones were clearly outruled by radiological imaging on both admissions and symptoms resolved quickly upon discontinuation of the antibiotic agent. We conclude therefore that Metronidazole can reasonably be identified as the only potential causative agent. 

This case provides the eighth report of Metronidazole-induced pancreatitis in the English language literature as shown in Table 1 [2]. All of the cases were reported in females and ran a benign course in continuity with most drug-induced pancreatitis [14].

There was a variable interval between exposure to Metronidazole and development of pancreatitis ranging from 12 hours [8] to 38 days [3], it should therefore be considered in patients presenting after minimal exposure to Metronidazole, in our case just 3 doses. A rechallenge is not to be recommended but this requires early recognition on the part of the clinician. 

The mechanism of action of Metronidazole-induced pancreatitis is unknown but the high incidence of concurrent illnesses known to induce acute pancreatitis makes a trigger role or cofactor role for the drug seem most likely [15]. One speculative mechanism of action is that under aerobic conditions Metronidazole may undergo redox cycling and yield hydrogen peroxide, superoxide, and other free radicals which are toxic to the pancreatic beta cells and induce pancreatitis [7]. It is interesting that due to good penetration of Metronidazole in the pancreas and the degree of coverage provided against the typical bacterial flora of the pancreas it is paradoxically indicated in the treatment of infected pancreatic necrosis [17].

Severe pancreatitis requiring hospital admission appears to be a rare adverse reaction [18]; however, in a recent population-based case-control study of 3083 cases and 30830 controls, Metronidazole was associated with a threefold increased risk of acute pancreatitis when used in combination with other drugs for H.Pylori eradication [19]. Like any drug-induced reaction, management of Metronidazole associated acute pancreatitis requires quick withdrawal of the offending agent and supportive care [12].

## 4. Conclusion

Pancreatitis is a disease with a wide variety of aetiologies, severity, complications, and outcomes. Metronidazole is commonly used in the hospital and community setting as it is a potent and effective treatment for many anaerobic infections. GI upset is a well- documented side effect of the drug. The incidence of severe Metronidazole-induced pancreatitis is relatively rare; however, it can cause serious morbidity in patients after minimal exposure, and identification of Metronidazole as the causative agent is the key to recovery. Early diagnosis, discontinuation of the drug, and supportive care will lead to a successful recovery in the majority of cases. Rechallenge should always be avoided.

## Figures and Tables

**Table 1:** 

Reference	Age (years)	Gender	Episode	Metronidazole to Pancreatitis (interval in days)	Indications for Metronidazole
[3]	29	Female	1	1	Postpartum unspecified vaginitis
			2	37	Unspecified vaginitis
[4]	63	Female	1	7	Crohn's disease
[5]	61	Female	1	4	Aspiration pneumonia
[6]	49	Female	1	3–5	Trichomoniasis
			2	Less than 1	Trichomoniasis
[7]	23	Female	1	8	Bacterial vaginosis
			2,3,4	3–7	Bacterial vaginosis
[8]	22	Female	1	Less than 1	Unspecified vaginosis
			2,3	1	Unspecified vaginosis
[2]	46	Female	1	8	Bacterial vaginosis
			2	8	Bacterial vaginosis
Our Case	25	Female	1	2	Endodontic abscess
			2	1	Endodontic abscess

**Table 2:** Progression of biochemical and haematological values during admission.

	Day 1	Day 2	Day 3	Day 4	Day 5	Day 6	Day 7
Amylase (27–131 U/L)	810	403	241	117	56	37	23
CRP (≤10 mg/L)	70	131	242	248	166	101	16
WCC (4–10 × 10^9^/L)	19.6	13.1	12.9	10.1	8.5	8.5	8.2
AST (15–41 IU/L)	20	24	19	16	12	15	14
Glucose (4.4–5.6 mmol/L)	5.4	4.9	3.7	3.5	4.9	4.2	3.8
Calcium (2.1–2.7 mmol/L)	2.16						
Albumin (35–50 g/L)	41	30	32	31	29	29	32
Urea (2.1–6.4 mmol/L)	3	2.2	1.6	1.4	1.5	1.1	2.7
Creatinine (39–91umol/ L)	56	53	57	61	50	53	52
Hb (13–17 g/dL)	13.3	11.2	11.2	11.9	11.1	10.9	11.8
